# Effectiveness and cost-effectiveness of routine third trimester ultrasound screening for intrauterine growth restriction: study protocol of a nationwide stepped wedge cluster-randomized trial in The Netherlands (The IRIS Study)

**DOI:** 10.1186/s12884-016-1104-8

**Published:** 2016-10-13

**Authors:** Jens Henrichs, Viki Verfaille, Laura Viester, Myrte Westerneng, Bert Molewijk, Arie Franx, Henriette van der Horst, Judith E. Bosmans, Ank de Jonge, Petra Jellema, The IRIS Study Group, Anneloes L. van Baar, Joke Bais, Gouke J. Bonsel, Jeroen van Dillen, Noortje T. L. van Duijnhoven, William A. Grobman, Henk Groen, Chantal W. P. M. Hukkelhoven, Trudy Klomp, Marjolein Kok, Marlou L. de Kroon, Maya Kruijt, Anneke Kwee, Sabina Ledda, Harry N. Lafeber, Jan M. van Lith, Ben Willem Mol, Marianne Nieuwenhuijze, Guid Oei, Cees Oudejans, K. Marieke Paarlberg, Eva Pajkrt, Aris T. Papageorghiou, Uma M. Reddy, Paul A. O. M. De Reu, Marlies Rijnders, Alieke de Roon-Immerzeel, Connie Scheele, Sicco A. Scherjon, Rosalinde Snijders, Marc E. Spaanderman, Pim W. Teunissen, Hanneke W. Torij, Tanja G Vrijkotte, Jos Twisk, Kristel C. Zeeman, Jun Zhang

**Affiliations:** 1 Department of Midwifery Science, AVAG and the EMGO+ Institute for Health and Care Research, VU University Medical Center, Van der Boechorststraat 7, A-511, 1081 BT Amsterdam, The Netherlands; 2 Department of Medical Humanities, EMGO+ Institute for Health and Care Research, VU University Medical Center, Van der Boechorststraat 7, 1081 BT Amsterdam, The Netherlands; 3 Department of Gynecology, Utrecht University Medical Center, Heidelberglaan 100, 3584 CX Utrecht, The Netherlands; 4 Department of General Practice, EMGO+ Institute for Health and Care Research, VU University Medical Center, Van der Boechorststraat 7, 1081 BT Amsterdam, The Netherlands; 5 Department of Health Sciences and the EMGO+ Institute for Health and Care Research, Vrije Universiteit Amsterdam, De Boelelaan 1085, 1081 HV Amsterdam, The Netherlands; 6 Department of Pediatrics, Emma Children’s Hospital, Amsterdam Medical Center, Meibergdreef 9, 1105 AZ Amsterdam, The Netherlands

**Keywords:** Intrauterine growth retardation, Perinatal outcome, Midwifery, Third trimester ultrasonography

## Abstract

**Background:**

Intrauterine growth retardation (IUGR) is a major risk factor for perinatal mortality and morbidity. Thus, there is a compelling need to introduce sensitive measures to detect IUGR fetuses. Routine third trimester ultrasonography is increasingly used to detect IUGR. However, we lack evidence for its clinical effectiveness and cost-effectiveness and information on ethical considerations of additional third trimester ultrasonography. This nationwide stepped wedge cluster-randomized trial examines the (cost-)effectiveness of routine third trimester ultrasonography in reducing severe adverse perinatal outcome through subsequent protocolized management.

**Methods:**

For this trial, 15,000 women with a singleton pregnancy receiving care in 60 participating primary care midwifery practices will be included at 22 weeks of gestation. In the intervention (*n* = 7,500) and control group (*n* = 7,500) fetal growth will be monitored by serial fundal height assessments. All practices will start offering the control condition (ultrasonography based on medical indication). Every three months, 20 practices will be randomized to the intervention condition, i.e. apart from ultrasonography if indicated, two routine ultrasound examinations will be performed (at 28–30 weeks and 34–36 weeks). If IUGR is suspected, both groups will receive subsequent clinical management as described in the IRIS study protocol that will be developed before the start of the trial.

The primary dichotomous clinical composite outcome is ‘severe adverse perinatal outcome’ up to 7 days after birth, including: perinatal death; Apgar score <4 at 5 minutes after birth; impaired consciousness; need for assisted ventilation for more than 24 h; asphyxia; septicemia; meningitis; bronchopulmonary dysplasia; intraventricular hemorrhage; cystic periventricular leukomalacia; neonatal seizures or necrotizing enterocolitis. For the economic evaluation, costs will be measured from a societal perspective. Quality of life will be measured using the EQ-5D-5 L to enable calculation of QALYs. Cost-effectiveness and cost-utility analyses will be performed. In a qualitative sub-study (using diary notes from 32 women for 9 months, at least 10 individual interviews and 2 focus group studies) we will explore ethical considerations of additional ultrasonography and how to deal with them.

**Discussion:**

The results of this trial will assist healthcare providers and policymakers in making an evidence-based decision about whether or not introducing routine third trimester ultrasonography.

**Trial registration:**

NTR4367, 21 March 2014.

## Background

Monitoring fetal growth and well-being is a major objective of prenatal care [1]. Intrauterine growth retardation has often been defined as failing to achieve a specific fetal biometric or estimated fetal weight threshold by a specific gestational age [2]. IUGR is a risk factor for adverse outcomes, including perinatal death, neonatal encephalopathy, neurodevelopmental impairments in childhood, and disease in adult life [3–6]. To be able to provide timely clinical management for these fetuses, sensitive screening procedures for the detection of IUGR are needed. In many Western countries, including the Netherlands, primary midwifery and/or obstetric care mainly consist of serial fundal height assessments to monitor fetal growth patterns. Yet, this approach is not very effective as it only detects about one fifth or fewer neonates being small-for-gestational-age (birth weight <10th percentile by gestational age) [7–9], which is troublesome as being small-for-gestational-age (SGA) is a common finding among perinatal deaths [10, 11].

An alternative approach to detect IUGR fetuses comprises routine third trimester ultrasonography. A recent prospective cohort study (*n* = 3977) demonstrated that routine third trimester ultrasonography using estimated fetal weight or abdominal circumference approximately almost tripled the detection rate of SGA neonates (sensitivity = 57 %) [12]. Routine third trimester ultrasound screening may have other benefits, including the detection of structural fetal abnormalities, e.g. craniospinal abnormalities and urinary tract abnormalities, which become manifest late in pregnancy [13]. However, a meta-analysis of 13 randomized trials among low-risk pregnancies (*n* = 34,980) did not reveal beneficial effects of third trimester ultrasound screening on primary outcomes of perinatal mortality, preterm birth less than 37 weeks, Caesarean section rates, and induction of labor rates [14]. Two major shortcomings have been identified in these previous trials [14, 15]. First, previous trials had methodological shortcomings, e.g., most trials were underpowered to detect clinically significant differences in severe perinatal outcomes, heterogeneity in number and timing of ultrasound scans, and contamination, i.e., ultrasound scans were often also conducted in the control group [14, 15]. Secondly, in many trials only the ultrasound screening procedure was described but not the use of subsequent management/intervention procedures when IUGR is suspected. If not coupled with an effective intervention, ultrasound screening alone cannot be clinically effective [12]. Moreover, ultrasound technology used in most previous trials is now outdated [14, 15]. Reviews on the effects of routine third trimester ultrasound screening on pregnancy and perinatal outcome concluded that a large-scale trial with adequate power is needed to address severe adverse perinatal outcomes and to examine long-term neurodevelopmental outcome in the offspring [14, 16].

Introducing a screening program can have negative consequences, such as unnecessary medical care [17]. Defining IUGR based on a certain cut-off, e.g., the lowest 10th percentile of estimated fetal weight, will probably not only lead to the detection of growth restricted fetuses but also to the classification of a group of ‘at risk fetuses’ who are constitutionally small and healthy. This may lead to unnecessary interventions, such as elective induction of delivery.

Moreover, additional third trimester ultrasonography may affect maternal emotions, either positively in that negative screening results may be reassuring, or negatively in that it may increase maternal emotional distress, i.e., anxiety or depressive symptoms. Women may experience higher levels of emotional distress, due to an (incorrect) indication of IUGR and be exposed to additional monitoring and obstetric interventions [18, 19]. Experiences of maternal emotional distress due to positive, unexpected or unclear findings based on fetal ultrasound screening may continue into the postpartum period, even when abnormal screening results have not been confirmed by subsequent examinations [20–22].

In the case of positive screening results routine third trimester ultrasonography may particularly be related to the experience of moral dilemmas by pregnant women and professionals performing ultrasonography. For example, parents may find this burdensome due to increased responsibility that comes with the fact that they have to choose for further examinations of the fetus (or not). Professionals may find it difficult how to decide how much and which information they should provide to women/parents and when to advice referral for further clinical management [20–23].

Despite the lack of evidence for its clinical effectiveness [14, 15], routine third trimester ultrasound screening is increasingly used in midwifery care in the Netherlands, which results in a considerable rise in health care costs. Few former studies evaluated the cost-effectiveness of ultrasound scans [24, 25]. Although costs of the ultrasound examination itself have previously been investigated, we know little about resulting costs (e.g., costs associated with subsequent counselling, follow-up examinations, and subsequent interventions). So far, only one previous trial, The Helsinki ultrasound trial, addressed this matter showing that one-stage second-trimester ultrasound screening is cost-effective in reducing perinatal mortality as compared to care as usual when taking all significant costs and effects into account [25]. Moreover, the cost-effectiveness of routine third-trimester ultrasound screening combined with serial fundal height measurements and clinically indicated ultrasonography as compared to care as usual (CAU), i.e. serial fundal height measurements and clinically indicated ultrasonography alone, has not been studied earlier.

In the Netherlands, at the moment no multi-disciplinary consensus exists concerning the screening for and clinical management of suspected IUGR. To be more specific, the current monodisciplinary Dutch guidelines of the Royal Organization of Midwifes in the Netherlands (KNOV) and of the Dutch Association of Obstetrics and Gynecology (NVOG) for IUGR detection or management have a different focus and do not fully align [26, 27]. This may lead to inconsistent approaches in the clinical management of suspected IUGR. Therefore, another key element of our study is the development of a consensus-based multidisciplinary protocol for the detection and subsequent management of suspected IUGR using a Delphi study.

### Research aims

The main aim of the (IUGR risk selection study) IRIS study is to assess the effectiveness and cost-effectiveness of routine third trimester ultrasound screening at 28–30 weeks and at 34–36 weeks of gestation in comparison with CAU in reducing severe adverse perinatal outcome among low-risk pregnant women through subsequent protocolized management. The research aims of the IRIS study are:

1. To evaluate whether routine third trimester ultrasound screening combined with subsequent protocolized management reduces severe adverse perinatal outcome as compared to CAU and subsequent protocolized management.
2. To evaluate whether routine third trimester ultrasound screening combined with subsequent protocolized management is cost-effective as compared to CAU and subsequent protocolized management.
3. To develop a multidisciplinary consensus‐based protocol for the detection and management of IUGR and to study professionals’ adherence to the protocol.
4. To examine whether routine third trimester ultrasound screening combined with subsequent protocolized management affects maternal prenatal and postnatal psychological functioning and infant neurodevelopment at age 6 and 24 months as compared to CAU and subsequent protocolized management.
5. To examine ethical dilemmas concerning positive, unclear, and unexpected findings and incorrect indication ofIUGR and to explore what professionals and women recommend regarding the handling of these ethical dilemmas.

## Methods/design

### Study design

The IRIS study is a nationwide stepped wedge cluster-randomized trial among 15,000 low-risk pregnant women receiving care at 60 midwifery practices in the Netherlands. The intervention entails routine third trimester ultrasound screening combined with serial fundal height measurements and clinically indicated ultrasonography, while the control condition entails CAU (serial fundal height measurements and clinically indicated ultrasonography only). In 1500 pregnant women derived from the entire study population a survey will be conducted to assess societal costs, maternal psychological functioning, maternal quality of life, and infant neurodevelopment. The exact design of the survey will be described in more detail later on.

Two sub-studies will be conducted as part of the IRIS study. In sub-study A, a Delphi study will be conducted to develop a multidisciplinary consensus-based protocol for the detection and subsequent management of IUGR in the Netherlands. Sub-study B will address ethical considerations of additional/routine third trimester ultrasound screening. These sub-studies will be described in more detail later on.

### Participants/eligibility criteria of participating midwifery practices

Practices will be eligible if all midwives are willing to follow the postgraduate registration training in the guideline ‘detection of IUGR’ of the KNOV [26]. Inclusion criteria for ultrasonographers that perform ultrasounds for women in the IRIS study are: 1) they have successfully followed the e-learning training for fetal biometry of the national medical e-learning education programs for medical students and professionals in the Netherlands (www.medischonderwijs.nl); 2) they possess a certificate for ultrasound anomaly screening (‘SEO’ certificate’) or will be judged as adequate performers of ultrasonography (based on 4 cases) by an IRIS study sonographer; 3) they use ultrasound equipment according to the standards of the NVOG [27]. Some midwifery practices perform ultrasound in their own practice; others refer to an ultrasound center.

Inclusion criteria for pregnant women are 1) receiving care in the participating midwifery practice at 22 weeks of gestation, having a singleton pregnancy and having no major obstetric or medical risk factors; and 2) having a reliable estimated date of delivery based on a dating ultrasound scan in line with NVOG guidelines or based on the first day of the last menstrual period [27].

### Recruitment and randomization

**Fig. 1:**
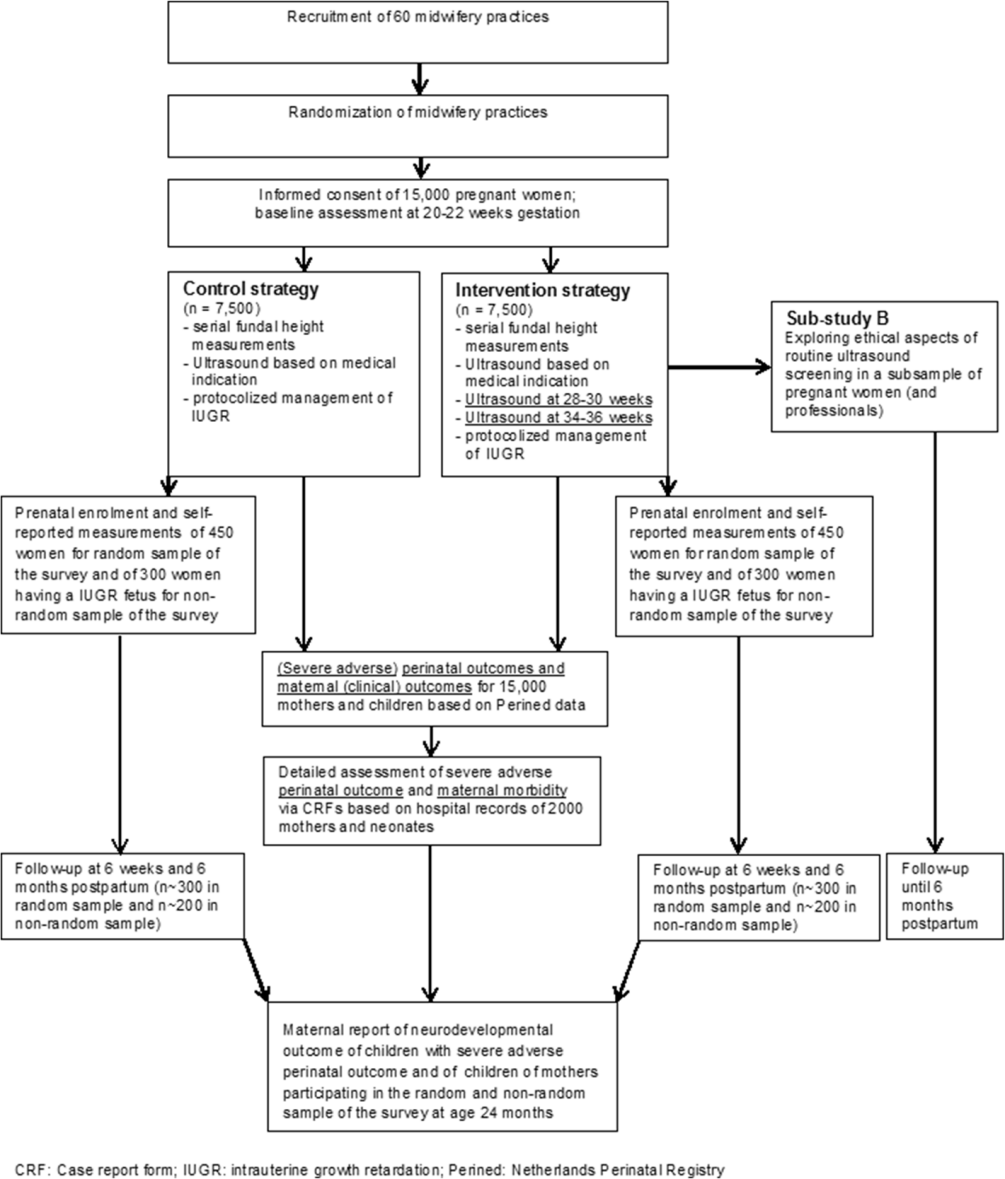
Flow chart study design

Figure 1 shows the flow chart of the study design. Midwifery practices will be informed about the IRIS study and invited to participate in the study via our nationwide Delphi study (sub‐study A). Other methods to invite midwifery practices for participation will include attending meetings of regional maternity care networks and the postgraduate training about the KNOV guideline [26], articles in national professional journals, and social media. A researcher will visit interested midwifery practices to provide information about the IRIS study, check whether the practice fulfils the inclusion criteria, and ask the midwives to sign a contract to demonstrate their commitment to the study protocol.

During the first consultation after the 20-weeks pregnancy ultrasound screening has been offered, eligible pregnant women will be given a trial information leaflet by their midwife. The midwife will obtain consent.

**Fig. 2:**
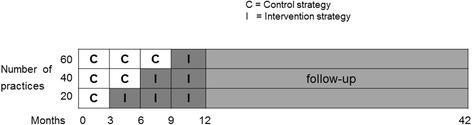
The stepped wedge design of the IRIS study. Pregnant women will be enrolled during months 1–12 at 20–22 weeks of gestation. All midwifery practices (*n* = 60) will start with the control condition providing care as usual. At intervals of 3 months, a third of all practices will change status from the control to the intervention condition, which means providing routine third trimester ultrasound screening at 28–30 weeks and 34–36 weeks of gestation. Postnatal follow-up will be conducted in months 18–42

Midwifery practices will form the unit of randomization. Randomization per practice rather than per midwife or woman minimizes contamination and maximizes contrast between the intervention and control group. As shown in Fig. 2, all midwifery practices (*n* = 60) will start in the control group. At 3 months intervals, a third of the midwifery practices will change status from the control to the intervention condition. To balance the number of women in the two conditions, practices will be stratified before randomization in large and small practices, with the average practice size as cut‐off (250 women per year). A stratified computer‐generated random sequence will determine the order in which practices change from control to intervention status. Randomization will be conducted by an independent statistician at 3 and 6 months after the start of baseline data collection and the recruitment of the first participating women.

### Care in the intervention and control group

Both the intervention group and the control group will receive the following standard elements of midwifery care: 1) serial fundal height measurements and clinically indicated ultrasonography in line with the KNOV guideline for the detection of IUGR [26]; 2) information about life-style factors that may influence fetal development, e.g., smoking and alcohol use; and 3) advice to report a reduction in fetal movements. When IUGR is suspected, both groups will receive subsequent management based on the consensus-based protocol that will be developed in sub-study A.

### Intervention

In the intervention group two routine third trimester biometry ultrasounds will be performed, the first at 28–30 weeks of gestation and the second at 34–36 weeks of gestation. Performing two ultrasound examinations enables detection of early and late fetal growth restriction and allows monitoring of fetal growth patterns, which may reveal decreased fetal abdominal growth velocity.

### Measures

#### Baseline characteristics of pregnant women and midwifery practices

To assess comparability of study groups and predictors of outcome, data on baseline characteristics of participating midwifery practices and participating pregnant women will be collected. Baseline characteristics of midwifery practices will include number of midwives working in the practice, number of clients per year, proportion of nulliparous and multiparous women, and rate of referral to secondary/tertiary care. Maternal baseline characteristics will include ethnic background, maternal age, educational level, height and weight, smoking, alcohol use, drug use, and work status during pregnancy.

#### Primary outcome – severe adverse perinatal outcome

The primary dichotomous clinical outcome of the main study will be a composite measure of severe adverse perinatal outcomes up to 7 days after birth defined as one or more of the following:

1. Antepartum, intrapartum or perinatal death occurring from 28 weeks of gestation onward
2. Apgar score <4 at 5 min after birth;
3. Coma, stupor or decreased response to pain up to 7 days after birth;
4. Asphyxia, defined as cord blood arterial base excess of less than minus 12;
5. Neonatal seizures defined as clonic movements which cannot be stopped by holding the limb, occurring on two or more occasions before 72 h of age;
6. Assisted ventilation for more than 24 h via endotracheal tube initiated within 72 h after birth;
7. Septicemia, ascertained by a positive blood culture;
8. Meningitis, ascertained by positive cerebrospinal fluid culture;
9. Bronchopulmonary dysplasia (BPD), defined as need for oxygen at a postnatal gestational age from 36 completed weeks as well as an X‐ray compatible with BPD;
10. Intraventricular hemorrhage, defined as grade 3 or 4 and diagnosed by cranial ultrasound or at autopsy
11. Cystic periventricular leukomalacia (PVL), diagnosed by cranial ultrasound or at autopsy showing periventricular cystic changes in the white matter excluding subependymal and choroid plexus cysts;
12. Necrotizing enterocolitis, defined as either perforation of intestine, pneumatosis intestinalis or air in the portal vein, diagnosed by X‐ray or surgery, or at autopsy.

#### Primary outcome - costs

Healthcare costs will include costs related to pregnancy-related healthcare use, including community midwife consultations, referrals to specialist care, ultrasound examinations, laboratory tests, CTG monitoring, hospital admission, interventions during labor, and admission to neonatal unit. Healthcare costs will be calculated using standard costs published in the Dutch costing guidelines [28]. Medication use will be calculated using prices of the Royal Dutch Society for Pharmacy.

Absenteeism and presenteeism at work (indirect costs) as reported by (pregnant) women will be assessed by the iMTA Productivity Cost Questionnaire (iPCQ) [29].

The friction cost approach will be used to estimate indirect costs using Dutch age and sex specific lost productivity costs [30, 31].

#### Secondary outcome measures- composite outcome

Two other dichotomous composite outcomes are defined as secondary outcomes. The first is spontaneous vaginal birth without intervention, i.e., a birth without any of the following interventions: 1) induction of labor other than amniotomy, 2) vacuum/forceps assisted birth, 3) Caesarean section; 4) augmentation of labor; and 5) pharmacological pain relief.

Secondly, another secondary dichotomous composite outcome is maternal perinatal morbidity/mortality, defined as the presence of one or more of the following: 1) maternal death within 42 days after giving birth, 2) hypertension, 3) pre‐eclampsia, 4) postpartum hemorrhage larger than 1000 mL, and 5) third or fourth degree perineal trauma.

#### Secondary outcomes – single outcomes

Single outcomes include the different elements of the composite primary outcome and the secondary composite outcome. Other secondary single outcomes will be neonatal mortality and severe morbidity between the 7th and 28th day after birth, congenital abnormalities, life threatening congenital conditions among neonates, non‐cephalic presentations (when labor started) in primary care, and place of birth. Mean birth weight, low birth weight, macrosomia, mean gestational age, and prematurity will also be single secondary outcomes.

Other secondary single outcomes, that will be reported by women participating in the survey (*n* = 1500), will include 1) maternal prenatal and postnatal quality of life (EQ‐5D‐5 L and one item of the short form health survey (SF-36) [32–34]); 2) maternal experience of continuity of healthcare and satisfaction with healthcare during pregnancy and delivery (Nijmegen Continuity Questionnaire (NCQ) and Pregnancy and Childbirth Questionnaire (PCQ)) [35, 36]; 3) maternal pre- and postnatal anxiety and pregnancy-specific anxiety (State and Trait Anxiety Inventory (STAI) [37], and the Pregnancy Anxiety Questionnaire-Revised (PRAQ-R)) [38]; 4) and pre- and postnatal depressive symptoms (Edinburgh (Postpartum) Depression Scale) [39, 40]; and 5) prenatal and postnatal maternal bonding, i.e., the emotional tie between the mother and the (unborn) child (Maternal Antenatal Attachment Scale (MAAS) and Maternal Postnatal Attachment Scale (MPAS)) [41, 42]. Moreover, other secondary single outcomes will be infant developmental milestones at age 6 months assessed with the Ages and Stages Questionnaire (ASQ) and toddlers’ behavioral problems and developmental milestones at 24 months measured via the Child Behavior Checklist (CBCL) and ASQ, respectively [43–45].

In a purposively selected subsample of pregnant women (n ~ 15) participating in the intervention group, semi-structured interviews will be conducted during late pregnancy to explore and better understand the role of third trimester ultrasound screening in the experience of maternal pregnancy-specific anxiety and maternal bonding.

#### Process measures

To evaluate the uptake of the intervention and the adherence to the consensus-based IRIS study protocol for the detection and management of IUGR, a number of process measures will be assessed, including the rate of women declining participation, the proportion of protocol violations, proportion of disagreements in primary outcomes based on reassessments by research assistants/nurses, and opinions of midwives on (in)effective elements of the intervention. Data on protocol adherence will be collected via standardized forms filled out by a researcher attending several multidisciplinary case evaluations or audits in case of perinatal deaths or severe adverse perinatal outcome. Protocol adherence will also be assessed via standardized case report forms filled out by research assistants/nurses using hospital records of a subsample of neonates and women (n ~ 2000) displaying perinatal/postpartum morbidity/mortality (for more detail see subsection data collection). Using a short questionnaire, we will evaluate community midwives’ experience of cooperation with healthcare professionals in secondary care in terms of the IRIS study protocol after the completion of the inclusion period.

### Data collection

#### Baseline questionnaire midwifery practice and women

Via a short questionnaire midwives will report characteristics of their midwifery practices based on the most recent Dutch national midwifery care report [46]. After enrolment, using short questionnaires, participating women and midwives will report maternal baseline characteristics, including data on demographics and anthropometrics.

#### Existing medical databases/registries

For the main study (*n* = 15,000), data will be extracted from the following existing databases: 1) the database of the Perinatal Registry of the Netherlands (Perined); 2) ultrasound centers’ databases; 3) hospital medical records of mothers and neonates if applicable. These databases will be used to collect data on (primary) clinical outcomes, obstetric variables, ultrasound scans, and care processes.

#### Survey

Two subsamples will be derived from the complete study population (see Fig. 1) for a detailed assessment of societal costs, maternal quality of life, maternal experience of healthcare during the perinatal period, maternal psychological functioning (maternal depressive symptoms, anxiety, and bonding), and infant neurodevelopment. These two subsamples comprise the following: (a) a random sample of 900 women (450 intervention and 450 CAU) who will be asked to complete online questionnaires around 22 weeks of gestation, at 32 weeks of gestation, and at 6 weeks, 6 months, and 24 months after estimated date of delivery (*n* = 15 per midwifery practice); and (b) a non-random sample comprising 600 women in whom IUGR is suspected by fetal ultrasonography (300 intervention and 300 CAU) and who will be asked to complete online questionnaires (*n* = 10 per midwifery practice) after the suspicion of IUGR during late pregnancy, at 6 weeks and 6 and 24 months postpartum. At the age of 24 months, we will ask mothers participating in the survey and mothers of toddlers having (a) severe adverse perinatal outcome(s) (see above for the definition of our primary clinical outcome) to report toddlers’ developmental milestones and behavioral problems.

For the survey, we will recruit 1500 women prenatally. Based on an expected non-response and drop-out rate of 33 %, we expect to collect complete follow-up data in 1000 women in the random (*n* = 600) and non-random sample (*n* = 400).

Women who will participate in the survey will give additional consent to participate in the survey and will receive an e-mail with a link to an online questionnaire at each measurement time point. Non-responding pregnant women will receive reminders via e-mail. To enhance participation of non-Dutch speaking women, the questionnaires will be translated into English. These questionnaire data will be collected through telephone interviews conducted by a researcher.

#### Hospital medical records

Using standardized case report forms research assistants/nurses will collect detailed information from approximately 2000 hospital medical records on healthcare utilization by and clinical outcomes of: 1) neonates who were referred to a pediatrician for neonatal admission, had a birth weight <5th percentile or a severe adverse perinatal outcome, e.g. Apgar score 5 min after birth <4, as indicated in the Perined database; and 2) women participating in the survey and being referred to a gynecologist/secondary care during the perinatal period, and women having a neonate who has been referred to a pediatrician, has a birth weight <5th percentile or a severe adverse perinatal outcome. The standardized case report forms will also be used to collect information on professionals’ adherence to the multidisciplinary consensus-based IRIS study protocol for the detection and clinical management of IUGR. By focusing on this group of neonates and women, we are able to efficiently collect data on severe adverse perinatal outcome, maternal perinatal morbidity, healthcare utilization, and protocol adherence which would not have been feasible in all 15,000 women.

### Statistical analyses

#### Sample size of the trial

Our sample size calculation was based on our primary dichotomous outcome, i.e. severe adverse perinatal outcome. Perined data suggest that the expected rate of severe adverse perinatal outcome in the source population, i.e. low-risk pregnant women in the Netherlands, is 1.54 %. Neither nationally nor internationally it has been agreed which degree of reduction in severe adverse perinatal outcome can be considered as feasible and clinically relevant. Therefore, the IRIS study group decided to aim at a reduction from 1.54 % to 1.0 % in the primary outcome. With 80 % power and a significance level of *p* < 0.05, 13,536 pregnant women should be included. Yet, due to the clustered design our sample size calculation also needs to take dependency of data into account. Pagel et al. (2011) estimated the intracluster correlation coefficients (ICCs) for a range of offspring perinatal outcomes using data from five community‐based cluster-randomized trials in three low‐income countries [47]. Five ICCs ranging from 0.0003 to 0.002 were reported for neonatal mortality [47]. We expect the ICC in the IRIS study to be much more similar to 0.0003 than to 0.002 for the following two reasons. First, the ICCs presented in the paper by Pagel et al. (2011) are based on prevalence rates of neonatal mortality ranging from 1.5 % to 5.9 % indicating a higher maximum rate than the rate of severe adverse perinatal outcome expected for the IRIS study, i.e. 1.54 % [47]. Since ICCs are expected to be related to low prevalences of the outcome of interest, we may assume the ICC in the IRIS study to be even lower than 0.0003. Second, participating midwifery practices will offer both CAU, and later on, routine third trimester ultrasound screening. Due to this, the variation in characteristics and practice management between the clusters, i.e. midwifery practices, will be reduced and, consequently, the size of the ICC will be lowered. Using the formula to correct for clustering [1 + (n‐1) * ICC] with *n* = 250, i.e. average cluster size in our study, the required sample size for the IRIS study is 14,547 women. Since not all pregnant women may be recruited exactly at 20–22 weeks of pregnancy, we decided to include a total of 15,000 pregnant women.

#### Effectiveness analyses

First, we will compare baseline characteristics of women participating in the intervention and control group using independent t-tests and chi-square tests. Second, we will compare baseline characteristics of drop-outs and completers by using independent t-tests and chi-square tests. Third, we will perform multiple logistic multilevel regression analysis to test the possible effect of routine third trimester ultrasound screening on severe adverse perinatal outcome. This analysis will be adjusted for possible clustering of observations at the level of midwifery practices and medical/biological, demographic and life-style related confounders. In all analyses, we will use an intention-to-treat approach and set the level of significance at *p* < 0.05.

### Economic evaluation

The economic evaluation aims to compare the costs generated by pregnant women receiving routine third trimester ultrasound screening versus those receiving CAU. Both a cost-effectiveness and cost-utility analysis will be conducted using different perspectives and time horizons.

#### Cost‐effectiveness and cost‐utility analyses

First, we will perform a cost‐effectiveness analysis from a healthcare perspective. The time horizon of this analysis ranges from 22 weeks of gestation to one week after the date of birth of the child. In this analysis, pregnancy related data derived from the Perined database of all 15,000 participating women will be used. Detailed cost data collected by research assistants/nurses from medical records of women participating in the random (*n* = 900) and non-random sample (*n* = 600) will be used to estimate the healthcare costs for the whole study population (*n* = 15,000) using Bayesian techniques in combination with Monte Carlo simulation. Incremental Cost‐Effectiveness Ratios (ICER) will be calculated by dividing the difference in healthcare costs by the difference in effects.

Second, we will perform a cost‐effectiveness analysis from a societal perspective. The time horizon of this analysis ranges from 22 weeks of gestation until 6 months after the estimated date of delivery. Again, pregnancy related data derived from the Perined database of all 15,000 participating women will be used in this analysis. Detailed cost data collected by research assistants/nurses from medical records, and self-report utilization and lost productivity data of women participating in the random (*n* = 900) and non-random (*n* = 600) sample will be used to estimate societal costs for the whole study population (*n* = 15,000) by applying Bayesian techniques in combination with Monte Carlo simulation. Furthermore, the combination of direct costs and indirect costs (based on maternal reports of absenteeism and presenteeism at (unpaid) work measured with the iPCQ [29]) will be related to the composite of severe adverse perinatal outcome to estimate the ICER.

Finally, a cost‐utility analysis from a societal perspective will be performed. The time horizon of this analysis ranges from 22 weeks of gestation until 6 months after the expected date of delivery. For this analysis, data of the 900 women in the random sample will be used. Quality‐Adjusted Life‐Years (QALYs) will be calculated based on the EQ‐5D‐5 L using the Dutch tariff) [48]. Incremental Cost‐Utility Ratios (ICURs) will be calculated by relating the difference in costs between the conditions to the difference in QALYs. In this analysis, missing cost and effect data will be imputed using multiple imputation based on the MICE algorithm developed by Van Buuren et al. (1999) [49]. Bias‐corrected and accelerated bootstrapping with 5000 replications will be used to estimate 95 % confidence intervals around cost differences and the uncertainty surrounding the ICERs and ICUR.

Both the cost‐effectiveness and cost‐utility analyses will be conducted using an intention‐to‐treat approach. For all analyses uncertainty surrounding the ICERs and ICUR will be graphically presented on cost‐effectiveness planes. Cost‐effectiveness acceptability curves will also be estimated using the net benefit framework [50]. Cost‐effectiveness acceptability curves will illustrate the probability that routine third trimester ultrasound screening for IUGR is cost‐effective (or not) as compared to CAU for a range of various ceiling ratios thereby demonstrating decision uncertainty.

### Description of sub-studies

#### Sub-study A

The first sub-study will concern a Delphi study aiming to develop a multidisciplinary protocol for the screening for, detection of, and subsequent management of IUGR in prenatal care in the Netherlands. Currently, the existing Dutch guidelines of the KNOV and NVOG for IUGR detection and/or management are not fully aligned and differ in scope potentially resulting in inconsistencies in clinical management between healthcare professionals [26, 27]. To facilitate multidisciplinary collaboration between professionals in primary and secondary/tertiary care and to develop uniform recommendations for IUGR screening and management participating panel members will receive structured online questionnaires in three rounds to achieve consensus about IUGR items. The questionnaires will address identified inconsistencies in the Dutch monodisciplinary KNOV and NVOG guidelines and will also be based on the evidence-based British guideline of the Royal College of Obstetricians and Gynaecologists (RCOG) [51]. This latter guideline addresses aspects of both screening for IUGR in the general population and additional examinations and management of IUGR. Panel members will be Dutch midwives, obstetricians, and sonographers, and national experts/researchers in IUGR and/or fetal biometry and monitoring. The multidisciplinary recommendations for IUGR screening and management resulting from this sub-study will be incorporated in the screening and management protocol of the IRIS study.

#### Sub-study B

In sub-study B we will investigate which ethical dilemmas concerning positive, unexpected, or unclear third trimester ultrasound findings and incorrect indication of IUGR the involved professionals and pregnant women experience and which recommendations they give for dealing with these ethical dilemmas in the future. For this purpose different qualitative methods will be used in an iterative way of working. First, an explorative literature study will be conducted to examine ethical dilemmas of pregnant women and professionals regarding decision-making, ultrasound screening procedures, and communication of ultrasound findings. Second, a purposively selected subsample of pregnant women (*n* = 32) participating in the intervention group will keep a textual, semi-structured diary for 9 months (from the beginning of late pregnancy until 6 months postpartum). In the diary study data on pregnant women’s experiences of the informed consent procedure, ultrasound screening, and ethical dilemmas (e.g., dealing with unexpected findings) will be collected. Third, among a subgroup of pregnant women (*n* = 10) individual semi-structured interviews will be held at 6 months postpartum to further explore thoughts and feelings about the third trimester ultrasound screening and related ethical dilemmas. Finally, two focus group interviews (*n* ~ 10) will be performed: one consisting of individually interviewed pregnant women and caregivers and the other group comprising multidisciplinary prenatal care professionals. Purposes of these focus group interviews will be: (a) deepen the understanding of the ethical dilemmas of pregnant women and caregivers, (b) validation of ethical dilemmas, and (c) establishing recommendations for future practice. Participants of sub-study B will be asked for written informed consent.

## Discussion

Sensitive measures to detect IUGR during late pregnancy and subsequent adequate clinical management of IUGR are important to decrease perinatal mortality and morbidity. This large-scale nationwide stepped wedge cluster-randomized trial will provide evidence whether or not routine third trimester ultrasound screening in combination with protocolized management is clinically effective and cost-effective as compared to CAU in reducing severe adverse perinatal outcome.

The current study has several strengths: it will fill crucial knowledge gaps in the domain of screening for and management of IUGR and is, therefore, expected to have important clinical implications. First, a major spin-off of the IRIS study is the development of a consensus-based multidisciplinary protocol for the detection and management of IUGR. This protocol can be used as starting point for the development of a multidisciplinary guideline for prenatal care of IUGR in the Netherlands. Second, as recommended in the recent Cochrane review by Bricker et al. (2015), the current study extends previous work by investigating the impact of routine third trimester ultrasound screening on maternal prenatal and postnatal psychological functioning and on long-term offspring neurodevelopmental outcome [14]. Third, we will identify pregnant women’s and professionals’ ethical dilemmas related to positive, unexpected and unclear findings during ultrasound screening. Based on these results recommendations for future practice can be formulated. Fourth, a methodological strength of our trial is its large sample size. In comparison to most previous studies [14], the current trial is adequately powered to examine whether third trimester ultrasound screening can reduce severe adverse perinatal outcome. Finally and importantly, our trial examines the cost-effectiveness and cost-utility of routine third trimester ultrasound screening among low-risk pregnancies as compared to CAU.

The results of the effectiveness and cost-effectiveness study will assist healthcare providers and policymakers in making an educated choice about whether or not introducing routine third trimester ultrasound screening in the Netherlands.

## Abbreviations

CAU: Care as usual
ICER: Incremental cost‐effectiveness ratios
ICUR: Incremental cost‐utility ratio
IRIS study: IUGR risk selection study
IUGR: Intrauterine growth retardation
KNOV: Royal Organization of Midwives in the Netherlands
NVOG: Dutch Association of Obstetrics and Gynecology
Perined: Perinatal Registry of the Netherlands
QALY: Quality‐adjusted life‐year
SGA: Small-for-gestational age
ZonMw: Netherlands Organization for Health Research and Development

## References

[1] Hall MH, Chng PK, MacGillivray I (1980). Is routine antenatal care worth while?. Lancet.

[2] Unterscheider J, Daly S, Geary MP, Kennelly MM, McAuliffe FM, O’Donoghue K (2013). Optimizing the definition of intrauterine growth restriction: the multicenter prospective PORTO Study. Am J Obstet Gynecol.

[3] Barker DJ (2006). Adult consequences of fetal growth restriction. Clin Obstet Gynecol.

[4] Bukowski R, Burgett AD, Gei A, Saade GR, Hankins GD (2003). Impairment of fetal growth potential and neonatal encephalopathy. Am J Obstet Gynecol.

[5] Murray E, Fernandes M, Fazel M, Kennedy SH, Villar J, Stein A (2015). Differential effect of intrauterine growth restriction on childhood neurodevelopment: a systematic review. BJOG.

[6] Unterscheider J, O’Donoghue K, Daly S, Geary MP, Kennelly MM, McAuliffe FM (2014). Fetal growth restriction and the risk of perinatal mortality-case studies from the multicentre PORTO study. BMC Pregnancy Childbirth.

[7] Hargreaves K, Cameron M, Edwards H, Gray R, Deane K (2011). Is the use of symphysis-fundal height measurement and ultrasound examination effective in detecting small or large fetuses?. J Obstet Gynaecol.

[8] Bais JM, Eskes M, Pel M, Bonsel GJ, Bleker OP (2004). Effectiveness of detection of intrauterine growth retardation by abdominal palpation as screening test in a low risk population: an observational study. Eur J Obstet Gynecol Reprod Biol.

[9] Sparks TN, Cheng YW, McLaughlin B, Esakoff TF, Caughey AB (2011). Fundal height: a useful screening tool for fetal growth?. J Matern Fetal Neonatal Med.

[10] Stacey T, Thompson JM, Mitchell EA, Ekeroma AJ, Zuccollo JM, McCowan LM (2011). The Auckland Stillbirth study, a case–control study exploring modifiable risk factors for third trimester stillbirth: methods and rationale. Aust N Z J Obstet Gynaecol.

[11] De Reu PA, Oosterbaan HP, Smits LJ, Nijhuis JG (2010). Avoidable mortality in small-for-gestational-age children in the Netherlands. J Perinat Med.

[12] Sovio U, White IR, Dacey A, Pasupathy D, Smith GC (2015). Screening for fetal growth restriction with universal third trimester ultrasonography in nulliparous women in the Pregnancy Outcome Prediction (POP) study: a prospective cohort study. Lancet.

[13] Chitty LS (1995). Ultrasound screening for fetal abnormalities. Prenat Diagn.

[14] Bricker L, Medley N, Pratt JJ (2015). Routine ultrasound in late pregnancy (after 24 weeks’ gestation). Cochrane Database Syst Rev.

[15] Bricker L, Mahsud-Dornan S, Dornan JC (2009). Detection of foetal growth restriction using third trimester ultrasound. Best Pract Res Clin Obstet Gynaecol.

[16] Alfirevic Z, Stampalija T, Gyte GM (2010). Fetal and umbilical Doppler ultrasound in normal pregnancy. Cochrane Database Syst Rev.

[17] Haynes B, Sacket DL, Gordon GH, Tugwell P: Clinical Epidemiology: how to do clinical practice research, 3rd edn. Philadelphia: Lippincott Williams and Wilkins; 2016

[18] Ahman A, Runestam K, Sarkadi A (2010). Did I really want to know this? Pregnant women’s reaction to detection of a soft marker during ultrasound screening. Patient Educ Couns.

[19] Viaux-Savelon S, Dommergues M, Rosenblum O, Bodeau N, Aidane E, Philippon O (2012). Prenatal Ultrasound Screening: False Positive Soft Markers May Alter Maternal Representations and Mother-Infant Interaction. PLoS One.

[20] Farsides B, Williams C, Alderson P (2004). Aiming towards “moral equilibrium”: health care professionals’ views on working within the morally contested field of antenatal screening. J Med Ethics.

[21] Lalor JG, Devane D, Begley CM (2007). Unexpected diagnosis of fetal abnormality: women’s encounters with caregivers. Birth.

[22] Watson MS, Hall S, Langford K, Marteau TM (2002). Psychological impact of the detection of soft markers on routine ultrasound scanning: a pilot study investigating the modifying role of information. Prenat Diagn.

[23] Gammeltoft T, Nguyen HT (2007). Fetal conditions and fatal decisions: ethical dilemmas in ultrasound screening in Vietnam. Soc Sci Med.

[24] Henderson J, Bricker L, Roberts T, Mugford M, Garcia J, Neilson J (2002). British National Health Service’s and women’s costs of antenatal ultrasound screening and follow-up tests. Ultrasound Obstet Gynecol.

[25] Leivo T, Tuominen R, Saari-Kemppainen A, Ylostalo P, Karjalainen O, Heinonen OP (1996). Cost-effectiveness of one-stage ultrasound screening in pregnancy: a report from the Helsinki ultrasound trial. Ultrasound Obstet Gynecol.

[26] Beentjes M, Roon-Immerzeel A, Zeeman K (2013). Opsporing van foetale groeivertraging KNOV-standaard [Detecting intrauterine growth restriction KNOV-standard].

[27] ederlandse Vereniging voor Obstetrie & Gynaecologie (2008). Foetale groeibeperking Versie 2.1 NVOG richtlijn [Fetal growth restriction Version 2.1 NVOG guideline].

[28] Hakkaart-van Roijen L, Van der Linden N, Bouwmans C, Kanters T, Tan SS (2015). Handleiding voor kostenonderzoek: Methodologie van kostenonderzoek en referentieprijzen voor economische evaluaties in de gezondheidszorg [Dutch manual cost research in the healthcare sector].

[29] Bouwmans C, Hakkaart-van Roijen C, Koopmanschap M, Severens H, Bouwer W (2013). Manual iMTA Productivity and Cost Questionnaire.

[30] Koopmanschap MA, Rutten FF (1996). A practical guide for calculating indirect costs of disease. Pharmacoeconomics.

[31] Hakkaart-van Roijen L, Tan SS, Bouwmans. Handleiding voor kostenonderzoek: methoden en standaard kostprijzen voor economische evaluaties in de gezondheidszorg. Geactualiseerde versie 2010 [Dutch manual for costing in economic evaluations]. Diemen, NL: CVZ; 2011.

[32] Herdman M, Gudex C, Lloyd A, Janssen M, Kind P, Parkin D (2011). Development and preliminary testing of the new five-level version of EQ-5D (EQ-5D-5 L). Qual Life Res.

[33] Ware JE, Sherbourne CD (1992). The MOS 36-item short-form health survey (SF-36). I. Conceptual framework and item selection. Med Care.

[34] Aaronson NK, Muller M, Cohen PD, Essink-Bot ML, Fekkes M, Sanderman R (1998). Translation, validation, and norming of the Dutch language version of the SF-36 Health Survey in community and chronic disease populations. J Clin Epidemiol.

[35] Uijen AA, Schellevis FG, van den Bosch WJ (2011). Mokkink HG, van WC, Schers HJ: Nijmegen Continuity Questionnaire: development and testing of a questionnaire that measures continuity of care. J Clin Epidemiol.

[36] Truijens SE, Pommer AM, van Runnard Heimel PJ, Verhoeven CJ, Oei SG, Pop VJ (2014). Development of the Pregnancy and Childbirth Questionnaire (PCQ): evaluating quality of care as perceived by women who recently gave birth. Eur J Obstet Gynecol Reprod Biol.

[37] Spielberger CD, Gorsuch RL, Lushene R, Vagg PR, Jacobs GA (1983). Manual for state-trait anxiety inventory.

[38] Huizink AC, Mulder EJ (2004). Robles de Medina PG, Visser GH, Buitelaar JK: Is pregnancy anxiety a distinctive syndrome?. Early Hum Dev.

[39] Cox JL, Holden JM, Sagovsky R (1987). Detection of postnatal depression. Development of the 10-item Edinburgh Postnatal Depression Scale. Br J Psychiatry.

[40] Pop VJ, Komproe IH, van Son MJ (1992). Characteristics of the Edinburgh Post Natal Depression Scale in The Netherlands. J Affect Disord.

[41] Condon JT (1993). The assessment of antenatal emotional attachment: development of a questionnaire instrument. Br J Med Psychol.

[42] Condon JT, Corkindale JC (1998). The assessment of parent-to-infant attachment: Development of a self-report questionnaire instrument. Journal of Reproductive and Infant Psychology.

[43] Achenbach TM, Rescorla LA (2016). Manual for ASEBA Preschool Forms & Profiles.

[44] Squires J, Twombly E, Bricker L, Potter LW (2009). ASQ-3 User's Guide.

[45] Steenis LJ, Verhoeven M, Hessen DJ, van Baar AL (2015). Parental and professional assessment of early child development: the ASQ-3 and the Bayley-III-NL. Early Hum Dev.

[46] Dijs-Elsinga J, Groenendaal F, van Huis AM, de Miranda E, Ravelle ACJ, Taminga P. Perined. Perinatale zorg in Nederland 2014 [Perined. Perinatal care in the Netherlands 2014].2015. Utrecht, NL, Perined.

[47] Pagel C, Prost A, Lewycka S, Das S, Colbourn T, Mahapatra R (2011). Intracluster correlation coefficients and coefficients of variation for perinatal outcomes from five cluster-randomised controlled trials in low and middle-income countries: results and methodological implications. Trials.

[48] Lamers LM, Stalmeier PF, McDonnell J (2005). Krabbe PF, van Busschbach JJ: [Measuring the quality of life in economic evaluations: the Dutch EQ-5D tariff]. Ned Tijdschr Geneeskd.

[49] Van BS, Boshuizen HC, Knook DL (1999). Multiple imputation of missing blood pressure covariates in survival analysis. Stat Med.

[50] Stinnett AA, Mullahy J (1998). Net health benefits: a new framework for the analysis of uncertainty in cost-effectiveness analysis. Med Decis Making.

[51] Guidelines Committee of the Royal College of Obstetricians and Gynaecologists (2013). The Investigation and Management of the Small-for-Gestational-Age Fetus RCOG Green-top Guideline No.31.

